# Neural Approach to Coordinate Transformation for LiDAR–Camera Data Fusion in Coastal Observation

**DOI:** 10.3390/s24206766

**Published:** 2024-10-21

**Authors:** Ilona Garczyńska-Cyprysiak, Witold Kazimierski, Marta Włodarczyk-Sielicka

**Affiliations:** 1 Department of Hydrography and Spatial Analyses, Faculty of Navigation, Maritime University of Szczecin, Waly Chrobrego 1-2, 70-500 Szczecin, Poland; i.garczynska@pm.szczecin.pl; 2 Marine Technology Ltd., 4/6 Roszczynialskiego St., 81-521 Gdynia, Poland; m.wlodarczyk@marinetechnology.pl

**Keywords:** camera–LiDAR fusion, USV, coastal monitoring, data fusion, neural networks

## Abstract

The paper presents research related to coastal observation using a camera and LiDAR (Light Detection and Ranging) mounted on an unmanned surface vehicle (USV). Fusion of data from these two sensors can provide wider and more accurate information about shore features, utilizing the synergy effect and combining the advantages of both systems. Fusion is used in autonomous cars and robots, despite many challenges related to spatiotemporal alignment or sensor calibration. Measurements from various sensors with different timestamps have to be aligned, and the measurement systems need to be calibrated to avoid errors related to offsets. When using data from unstable, moving platforms, such as surface vehicles, it is more difficult to match sensors in time and space, and thus, data acquired from different devices will be subject to some misalignment. In this article, we try to overcome these problems by proposing the use of a point matching algorithm for coordinate transformation for data from both systems. The essence of the paper is to verify algorithms based on selected basic neural networks, namely the multilayer perceptron (MLP), the radial basis function network (RBF), and the general regression neural network (GRNN) for the alignment process. They are tested with real recorded data from the USV and verified against numerical methods commonly used for coordinate transformation. The results show that the proposed approach can be an effective solution as an alternative to numerical calculations, due to process improvement. The image data can provide information for identifying characteristic objects, and the obtained accuracies for platform dynamics in the water environment are satisfactory (root mean square error—RMSE—smaller than 1 m in many cases). The networks provided outstanding results for the training set; however, they did not perform as well as expected, in terms of the generalization capability of the model. This leads to the conclusion that processing algorithms cannot overcome the limitations of matching point accuracy. Further research will extend the approach to include information on the position and direction of the vessel.

## 1. Introduction

Coastal monitoring is an important aspect of people’s activities for both environmental and security reasons. It is especially important in harbor areas, which are dynamically changing and intensely exploited environments. Suitable observation for various purposes can be performed with fixed cameras; however, this solution has limitations regarding covered areas. Therefore, the use of unmanned vehicles is increasingly popular for this purpose. UAVs (unmanned aerial vehicles) are an especially popular solution, providing a bird’s eye view of the area. The article [1] discusses the use of remote sensing and LiDAR technology in assessing marine and coastal areas. The authors elaborate on methods for data acquisition and processing in the context of analyzing seabed morphology and environmental changes. The study [2] explores the fusion of heterogeneous data from synthetic aperture radar (SAR), optical satellite imagery, and airborne LiDAR for effective surface water detection. The results demonstrate that using a high-resolution LiDAR digital elevation model significantly enhances the accuracy of water classification, reducing uncertainty in detection compared to single-polarization SAR methods, and highlights the benefits of integrating various remote sensing techniques in natural landscapes. The authors of [3] emphasize the significance of utilizing a digital elevation model (DEM) derived from LiDAR, which greatly enhances the accuracy of water classification and reduces detection uncertainty compared to methods based solely on synthetic aperture radar (SAR).

An interesting alternative to this is to use USV (unmanned surface vehicle), providing a unique side view of the coastal/harbor zone. Additionally, the system can be supplemented with LiDAR (Light Detection and Ranging), which can provide a digital point cloud and, after processing, a 3D models of the area. The system then is similar to the ones used on autonomous cars; however, it is mounted on a different platform and used for other purposes, facing very different challenges. The article [4], on the other hand, discusses methodologies for conducting bathymetric and photogrammetric measurements in coastal areas using unmanned aerial vehicles (UAVs) and unmanned surface vehicles (USVs), though without the use of LiDAR as a sensor.

The integration of camera and LiDAR data eliminates many issues associated with single sensor data. LiDAR provides accurate metric data, while imagery allows us to visualize the surrounding environment and identify features. Thus, data from both sources provide different kinds of information, which, after fusion, can provide a comprehensive picture of the area and situation. Independent processing of data from sensors should be supported by fusion techniques to achieve a synergy effect and common information. For coastal mapping, especially in harbor areas, an additional challenge is caused by limited possibilities for data acquisition at the desired close distance to the shore, due to the frequent movement of vessels in port areas. LiDAR has restrictions related to the range of the transmitted laser beam, while the camera allows precise visualization of the terrain. The integration of these data makes it possible to perform advanced analyses of the study area.

Various strategies for the challenge of fusing camera and LiDAR data are discussed in the literature, as outlined in the next section. Many researchers emphasize the difficulty of data integration due to the lack of time synchronization, sensor calibration, the lack of a stable structure on which the sensors are mounted, and ensuring that both sensors remain in a horizontal plane [5,6,7,8]. Different filtering algorithms and data processing methods, as well as fusion and calibration techniques, are used for this task. A review of them is given in Section 2, presenting the background for our research. Most approaches make use of the geometrical properties of the measurement system and the sensors themselves, which are the basis for fusion. Most of the approaches also focus on an integrated and calibrated system of sensors on a fixed platform in a controlled, non-floating environment. In this aspect, the research provided in this paper gives a novel contribution to the field.

This article presents an innovative approach to the subject, addressing the requirements of data fusion from sensors on a dynamic floating platform such as an unmanned surface vehicle. The unique environment in which the tests were conducted often presents challenges in meeting the necessary conditions for correct data fusion. In such a situation, information about vehicle movement is essential. This can be provided by additional sensors, yet it requires additional processing techniques (such as signal filtering and integration) included in fusion algorithms. Therefore, to overcome this issue, in this paper, we propose a method that involves creating matching points using expert knowledge. Corresponding points are selected manually from the LiDAR point cloud and images to create training sets for the neural networks. The trained networks are then used to fuse the image with the point cloud. This allow focusing on the training process rather than on finding suitable parameters for filtration and signal processing. Thus, the method is based on data collected from both sensors, which then had to be independently processed. Homologous points were used to train artificial intelligence models as well as for numerical transformations. This approach allowed us to effectively compare the proposed neural methodology with numerical methods.

The data used in the study wer collected by the autonomous surface vehicle called Hydrodron. The vehicle can operate in autonomous mode, following a planned trajectory, or in remote control mode, which is particularly useful in challenging navigational situations and areas that are inaccessible or difficult for larger manned vessels. The Hydrodron is equipped with navigation sensors, hydrographic sensors, and additional sensors for monitoring the surrounding environment. Data from video cameras are also recorded. Data acquired from the LiDAR and camera are stored on onboard industrial computers. The research used data collected during a project entitled *Innovative multidimensional and multitemporal coastal zone monitoring system using an autonomous unmanned vessel*. As part of the project, a multidimensional and multitemporal coastal zone monitoring system will be created. Modern sensors were employed for data acquisition: MBES (multibeam echosounder) and side-scan sonar for underwater data, and a LiDAR and camera for terrestrial data. The system integrated data from various sensors, creating a comprehensive multidimensional and multitemporal database of the coastal zone. All sensors and system components were mounted on a single vessel. Data were collected during one route and then integrated into a unified dataset. The benefits of this system include monitoring the coastal zone, inventorying the condition of navigational markings, waterways, shoreline, bottom rubble movements, quayside inspections, fairway capacity checks, and updating flood hazard maps. The system would enable an assessment of how measured and visualized changes impact the surrounding environment.

## 2. Background

This section provides an overview of the following research topics discussed in this paper: the most common applications of LiDAR and camera data fusion (Section 2.1), the sensor calibration problem (Section 2.2), LiDAR and camera fusion for coastal mapping (Section 2.3), and the use of AI (artificial intelligence) in data fusion (Section 2.4).

### 2.1. LiDAR and Camera Data Fusion

The research analysis provides valuable insights into the fusion of camera and LiDAR data, including calibration methods and the impact on perception systems for applications such as autonomous vehicles and robotics. The main problem with cameras is that typical consumer-grade RGB-D cameras often come with coarse intrinsic and extrinsic calibrations, which generally do not meet the accuracy requirements of many robotics applications, such as highly accurate three-dimensional (3D) environment reconstruction and mapping, high precision object recognition, localization, etc. [9].

Another very important aspect is that camera-supplied data, which is actually an image, lacks depth information and is susceptible to noise and interference, particularly influenced by weather conditions. Additionally, data quality depends on the time of day because of the camera’s sensitivity to light. LiDAR, on the other hand, despite providing accurate metric information, has limitations related to extracting the textural properties of objects due to the often-sparse distribution of point clouds [10]. This sensor is also dependent on weather and lighting conditions.

In fusion scenarios, a common challenge is the limitation associated with achieving precise alignment of data from these two modalities, which results from spatiotemporal synchronization problems [11]. It is important to remember the crucial issue of angular mismatch, which can lead to significant absolute deviation at longer distances. The research is therefore often undertaken on calibration methods for the two sensors. Based on a review of the existing literature, it can be concluded that the problem is so complicated that practical step-by-step instructions on how to integrate data from the sensors discussed above are not widely available, especially for floating platforms. It should be noted that in most articles, the LiDAR and camera were in a stable position during data acquisition (for example, in automotive solutions), ensuring that the point cloud and image were collected simultaneously.

The topic related to the fusion of camera and LiDAR data has become widely utilized, particularly in the field of mobile robots. Simultaneous localization and mapping (SLAM), based on these sensors, is currently a prominent research area attracting the attention of many scholars. LiDAR and camera sensors through their ability to detect both geometric and textual information about the environment are the most commonly used sensors in mobile robots for robot navigation including mapping and tracking [12]. The fusion of camera and LiDAR involves combining camera images and LiDAR point clouds to improve object detection and ranging in a real-time driving environment [13]. Additionally, integrating LiDAR systems with cameras enhances the environmental perception capabilities of autonomous platforms like unmanned vehicles and robots [14]. The fusion of camera and LiDAR data is, therefore, increasingly being used to improve the accuracy of perception systems across various applications, including autonomous vehicles and intelligent monitoring [15]. This usefulness stems from the complementary nature of camera and LiDAR sensors, which provide information that increases the reliability and accuracy of the entire perception system [16]. While camera images offer fine-grained texture and color information in 2D space, LiDAR captures precise and distant measurements of the surrounding environment [11]. Zheng et al. [17], inspired by the complementary characteristics of LiDAR and camera sensors, propose a new end-to-end learnable framework named point-image fusion network (PIFNet) for integrating LiDAR point cloud and camera images collected from the car. To resolve the problem of inconsistency in localization and classification, they designed an Encoder–Decoder Fusion (EDF) module to extract image features effectively while maintaining the fine-grained localization information of objects. In a study by Liu et al. [18], a localization and mapping scheme named LVI-fusion, based on multi-sensor fusion of camera, LiDAR, and inertial measurement units (IMU), is proposed. Different sensors have different data acquisition frequencies. To address the issue of time inconsistency in heterogeneous sensor data tight coupling, the time alignment module is used to align the timestamp between the LiDAR, camera, and IMU sensors.

Due to the increased application of LiDAR and cameras in recent years, fusion technology has been continuously developed. Time synchronization and spatial registration, prerequisites for data fusion from multiple sources, have been investigated by various researchers; for example, by [5,6,7,8]. The authors of [19] presented a method for fusing data captured by rigidly mounted LiDAR and cameras, demonstrating efficient and accurate fusion of a 3D LiDAR point cloud with 2D color image data. The integration of LiDAR and photogrammetric data has been proposed for platform georeferencing and mapping, showing potential for environmental monitoring and mapping applications [20]. Researchers in [21] have demonstrated that combining LiDAR data with optical very high resolution (VHR) data improves the analysis and monitoring of specific areas of interest, such as exploring external environments and extracting building features from urban areas. It should be noted that all the above-described research is applicable to the acquisition of data on land. In the cases described, the sensors are mounted on the car, which omits many problems encountered on USVs traveling on the water surface. Another group of publications shows the applications on UAVs (unmanned aerial vehicles). In the publication [22], the authors present LiDAR integration onto UAVs, providing a cost-effective means to acquire remotely sensed images with unprecedented spatial detail. This integration provides physiochemical and structural insights into the environment, particularly for monitoring sensitive environments like swamp vegetation in longwall mining areas. In paper the paper by Steenbeek et al. [23], the real-time capabilities of a commercial, inexpensive UAV (DJI Tello) for indoor mapping are investigated. The study aims to assess its suitability for quick mapping in emergency conditions to support first responders (FR) during rescue operations in collapsed buildings. In the paper by Xu et al. [24], an unmanned aerial system that integrates three cameras (RGB, multispectral, and thermal) along with a LiDAR sensor is developed. They implemented data acquisition software supporting recording and visualization to run on the robot operating system.

The fusion of LiDAR and camera data has various applications in 3D object detection, enhancing the performance of 3D detection systems for autonomous platforms such as unmanned vehicles and robots [25]. It is also used for object detection, classification, localization, and mapping in autonomous vehicles, contributing to improved environmental perception and recognition abilities [26]. Kumar et al. [27] presented a method to estimate the distance (depth) between a self-driving car and other vehicles, objects, and signboards on its path using an accurate fusion approach. Based on the geometrical transformation and projection, low-level sensor fusion was performed between a camera and LiDAR using a 3D marker. Additionally, the real-time fusion of raw data from LiDAR and camera sensors is applied for road segmentation in autonomous vehicle technology, leading to increased efficiency and affordability [28]. It is important to mention, on the other hand, the possible challenges and problems. High computational complexity and system accuracy as well as stability susceptible to incorrect depth matching are challenges faced by existing fusion algorithms [29]. Other challenges include the difficulty of finding reliable correlations between data of very different characteristics, such as geometry versus texture and sparse versus dense data [30].

The fusion of camera and LiDAR technologies improves object detection and recognition in autonomous vehicles by combining complementary information from both sensors. However, there are challenges and limitations in integrating camera and LiDAR fusion in real-time applications. Current research trends focus on the development of fusion cameras and LiDAR systems for environmental sensing and mapping.

### 2.2. The Problems with Calibration

Camera–LiDAR data fusion requires precise sensor calibration for high accuracy in perception systems. Abbasi et al. [15] presented a step-by-step calibration process implemented in MATLAB to determine the correspondence between a camera and LiDAR, making it easier for novice researchers to adapt for their applications. Wang et al. [14] presented a comprehensive review of external calibration developments between LiDAR and cameras, considering target requirements and geometrical constraints such as lateral and longitudinal dimensions. External calibration was also addressed by Duan et al. [31], who established the relative positional relationship between stereo and LiDAR 3D cameras. In their contribution, Grammatikopoulos et al. [32] presented a simple and intuitive approach for estimating the exterior (geometrical) calibration of a LiDAR instrument with respect to a camera, along with their synchronization shifting (temporal calibration) during data acquisition.

Similarly, authors Pandey et al. [33] optimized extrinsic calibration by maximizing mutual information between the colormap and the image. Scaramuzza et al. [34] and Levinson and Thrun [35] detected and extracted 3D edges from the point cloud using laser beam depth discontinuity. The 3D edges were then back-projected onto the 2D image plane to calculate the residuals. However, the accuracy of this method is limited by the estimation of edges, as the laser points do not strictly fall on the depth discontinuity margin.

Dong et al. [36] proposed a method for the joint calibration of LiDAR and camera based on dual constraints of edge corners and interior points, achieving high calibration accuracy. Liu et al. [37] proposed a novel approach for automatic extrinsic calibration between LiDAR and a camera, significantly improving data fusion accuracy. The proposed method aligns 3D points in the LiDAR frame with pixels in the image frame with 47.59% higher accuracy than the state of the art.

Another approach to the topic is presented by Yuan et al. [38], who proposed a method for automatic external calibration of high-resolution LiDARs and RGB cameras in targetless environments, achieving pixel-level accuracy by aligning natural edge features from both sensors. This method does not need to be calibrated based on a strict target, such as checkerboards, which are commonly used [39,40,41]. Xie et al. [42] presented the pixel and 3D point alignment (PPA) method, which directly calculates the alignment between 3D points and pixels without the need for camera parameters and calibration of the coordinate transformation matrix. This method enhances accuracy and robustness against noise in the calibration process. Zhou et al. [43] proposed a targetless extrinsic calibration method for monocular cameras and LiDAR sensors that uses pose transformation to establish data association across different modalities. This approach transforms the calibration problem into an optimization problem within a visual SLAM system without the need for overlapping views.

Calibration of the combined measurement system is shown as an important factor affecting the accuracy of fusion. However, calibration procedures and algorithms are usually complicated and time-consuming, especially since that they have to be done for each and every system separately.

### 2.3. LiDAR and Camera Fusion for Coastal Mapping

LiDAR, as a high-end depth sensor, provides a long outdoor range, accurate measurements, and system robustness [44]. It provides 3D spatial information and accurate depth perception [45]. It offers a long detection range and relatively low cost [46,47]. It produces point cloud data with high resolution but lacks texture and color information, which is why the idea is to combine it with a camera to obtain the missing data.

As a review of the existing literature shows, the integration of data from these sensors has found applications also in shoreline mapping. While optical imaging has limitations for shoreline mapping, airborne LiDAR data can provide more accurate topographical information. However, challenges in data availability and extraction techniques have also been identified [45]. An example of a robust LiDAR-camera fusion pipeline in unified bird’s eye view (BEV) space can be found in [48]. It has been developed to address sensor malfunctions and ensure stable performance regardless of single-modality data corruption. In a research by Lee et al. [49] and Song et al. [50], airborne LiDAR has been used for monitoring coastal erosion, providing topographical data for the intertidal zone, and effectively and quantitatively monitoring topographical changes. In another example, integrated LiDAR-imagery systems have been applied to various environmental surveys, including the detection of distributed pollution and discrete targets in the water column, as well as identifying bottom disorders in coastal zones [51]. LiDAR technology has been used to examine coastline changes, contributing to the accurate determination of coastal lines, a fundamental element of Integrated Coastal Zone Management [52]. An interesting example of using LiDAR for monitoring the coastal zone is the so-called bathymetric LiDAR, which can be used to provide a joint topo-bathymetric model [53]. This is again a bird’s eye-view perspective. An interesting example of camera image processing from a platform perspective can be found in [54]; however, it lacks fusion with LiDAR.

However, while LiDAR data provides high-resolution topographic and bathymetric mapping, it may have insufficient resolution for tasks like object recognition and feature extraction. The time required to analyze data and manage massive point clouds systematically, as well as the need to eliminate irrelevant data, poses challenges in integrating LiDAR data for coastal monitoring [55]. Additionally, high-power laser systems with wide-aperture optics are crucial for optimal LiDAR performance in turbid water conditions, presenting challenges in environments with varying water types [56]. The depth penetration of LiDAR sensors is limited by water clarity, and changing weather conditions can affect the effectiveness of the surveying technique [57]. Furthermore, the cloudy and rainy environment in coastal zones poses challenges for high-quality optical imagery and continuous monitoring of land use/cover, soil quality, vegetation, coastal line, and water color [58]. Therefore, camera data are also supplemented with additional acoustic models to provide complex observations, as, for example, in [59] or [60].

It should be mentioned that advances in sensor design and data analysis techniques have made remote sensing systems, including LiDAR and cameras, suitable for monitoring coastal ecosystems and their changes, such as beach profiles and submerged aquatic vegetation [61]. An innovative underwater 360° Pulsed Laser Line Scanner (PLLS-360°) system, integrated with an AUV equipped with an interferometric side scan sonar, allows for LiDAR–sonar data fusion, enhancing mapping of the seafloor, water column, and water surface simultaneously [62]. The authors in [63] took measurements from sensors installed on an autonomous, unmanned hydrographic vessel and proposed a data fusion mechanism, to create visualizations using modules both underwater and above the water surface. This fusion involves key-point analysis on classic images and sonars, augmentation/reduction of point clouds, fitting data, and mesh creation. A review of the literature in this research direction indicated a wide application of such data integration, primarily focusing on the acquisition of aerial photographs and the use of green LiDAR in research related to bottom and water analysis.

To date, coastal zone studies have focused on data integration using UAVs or LiDAR, which provided data from a top-down view. On the other hand, similar fusion solutions for land-based vehicles (e.g., autonomous cars) are known, where the stability of the vehicle is ensured or they are equipped with specialized equipment that offers a preconfigured environment for measurements.

Recently developed sensor fusion solutions are also available on the market, such as those provided by RIEGL. These laser scanners are supplemented with position sensors, GPS (global positioning system), and IMU (inertial measurement unit). The addition of the cameras complements the LiDAR data. As a result, users receive a finished colored point cloud, which serves as a starting point for analysis. However, this solution involves high costs and the methods and algorithms used in the fusion process themselves are not published.

The proposed research approach is original and novel, because, as the literature review shows, the methods for integrating information in the coastal zone that consider lateral perspective data acquisition based on area matching are not currently available. In addition, the dynamics of the vessel have been taken into account, which is not a common solution.

### 2.4. The Use of AI in Data Fusion

The fusion of LiDAR and camera data using AI has been explored in various studies. One approach involves fusing distance information and camera data to navigate in indoor environments, thereby reducing errors generated by individual techniques [64]. Another method employs a fusion algorithm to achieve accurate detection and tracking of road users in automated vehicles, demonstrating real-time applicability [65]. Additionally, a novel fusion framework, BEVFusion, has been proposed, which does not rely on LiDAR data input. This framework addresses the limitations of previous methods and surpasses state-of-the-art approaches under both normal and robustness training settings [66].

One of the major challenges in fusing LiDAR and camera data using AI is again the need for accurate extrinsic calibration between the two sensors, which forms the foundation of multi-modality data fusion. Another challenge is the accumulation of errors in traditional methods, which can lead to degraded operation and performance. Furthermore, risk factors associated with both LiDAR and camera sensors, such as transparency and reflections, can reduce data quality and impair performance [67].

Future research directions in the fusion of LiDAR and camera data using AI include the development of deep learning-based calibration methods to improve accuracy and robustness, as well as online calibration in natural scenes. The focus will be on improving accuracy, increasing robustness, automating the calibration process, and establishing a unified verification standard to further enhance the calibration process and its applicability in various domains [68]. Additionally, development trends are expected to focus on long-distance imaging in adverse environmental conditions, high-resolution fast 3D imaging, and high-performance color LiRAI (light ranging and imaging) [69].

It is also worth mentioning that the application of convolutional neural networks (CNNs) has been also widely used in recent years for LiDAR and image processing, mostly in the context of object recognition and classification, but also for fusion. CNNs are used to integrate LIDAR data (typically in the form of point clouds) with RGB images from cameras, enabling more effective object recognition and a better understanding of scene context. Examples of networks used for such fusions include PointNet (for processing point clouds) and ResNet (for processing images), which can be combined to create a model capable of processing both visual and spatial data.

In the article [70], multi-view 3D networks (MV3D) are proposed as a sensory-fusion framework that takes LIDAR point clouds and RGB images as input and predicts oriented 3D bounding boxes. They are based on the VGG16 network (being a type of convolutional network). The proposed network efficiently generates 3D candidate boxes from the bird’s eye view representation of the 3D point cloud. A deep fusion scheme has been developed to combine region-wise features from multiple views and enable interactions between intermediate layers of different paths. The primary focus of this paper is on 3D object detection utilizing both LIDAR and image data. The main idea for using multimodal information involves performing region-based feature fusion.

The article [71] utilizes CNNs for processing LiDAR data and integrating it with images for object detection. In this work, PointPillars is proposed as a novel encoder that employs PointNets to learn a representation of point clouds organized in vertical columns (pillars). This approach efficiently leverages the spatial structure of point clouds, enabling faster and more accurate object detection by combining information from both LiDAR and image data.

The paper [72] provides a detailed review of various deep architectures and models, highlighting the characteristics of each specific model. The focus is mainly on the application of deep learning architectures to three major areas: wild animal detection, small arm detection, and human detection. The comprehensive analysis examines the strengths and weaknesses of different models in these applications, offering insights into their performance and effectiveness in real-world scenarios.

For semantic segmentation and classification of 3D LiDAR point clouds, several methods leveraging deep learning have been explored. Techniques such as PointNet and PointNet++ are commonly utilized for directly processing raw point clouds by learning point-wise features, which enables efficient segmentation and classification.

The article series “3D Point Cloud Analysis” [73] discusses both traditional and modern deep learning methods for point cloud analysis, encompassing segmentation, classification, and registration. This book provides a comprehensive analysis of deep learning methods, including those based on PointNet, and presents experimental results that compare different approaches for point cloud processing, including semantic classification.

Another example can be found in [74], where CNN architecture is used to perform semantic segmentation of LiDAR data by slicing the 3D point cloud into 2D grids, ultimately providing 3D segmentation.

### 2.5. Conclusion and Proposed Approach

In conclusion, the review shows that the fusion of LiDAR data and camera data is a prominent topic, particularly in autonomous vehicle navigation and environmental monitoring. Advancements in the use of AI can be seen, but mostly for sensor data processing. However, challenges remain in the fusion process, such as accurate extrinsic calibration and addressing risk factors associated with sensor data quality. The applications of this fusion have implications for autonomous vehicles, robotics, and environmental perception. Future research directions aim to address these challenges and further enhance the accuracy and robustness of the fusion process. Additionally, in the case of dynamic floating platforms like autonomous surface vehicles, issues related to platform movements on water can arise, impacting the fusion process and quality.

To overcome challenges associates with calibration of sensors and the entire system, as well as to make the process platform-independent, we propose in this research using coordinates transformation based on point matching algorithms as a base for the fusion. Such approach shifts computational burden to post-processing part making the task more information-fusion than typically used sensor fusion. Some similarities to coordinate transformation known in cartography and to spatial adjustment met in geoinformatics and spatial analysis can be met here. Therefore, use of the methods known from these are-as is proposed.

The above analysis shows that CNNs and other deep learning structures have been used for complex tasks related to LiDAR and image analysis and fusion. The problem of point matching presented in this article may not be as complex, so simpler structures will be tested first. The process in this paper does not include automatic segmentation, and initially, we assume the manual selection of points. Segmentation and classification could ease this process, but on the other hand, it may result in removing some data, which may lead to some inconsistencies. Therefore, at the moment, we have decided not to use deep structures to simplify the process in this research. However, future approaches may assume more feature-based fusion, including semantic segmentation and classification, at which point more complex structures will be tested.

## 3. Materials and Methods

The data or information fusion process involves combining two or more entities from various sources based on certain rules to achieve a better (more accurate or more complex and comprehensive) result. In the case of a LiDAR system, we have an accurate geometric model, lacking identification. In the case of the camera, we can identify objects, but its ability to position and measure them is limited. Thus, the fusion can provide a comprehensive and geometrically accurate model of the shore side.

In this section, we formulate the problem to be solved (Section 3.1) as well as describe the methods that will be used for this, presenting the proposed approach (Section 3.2, Section 3.3 and Section 3.4). In the later sections, we provide details about the research experiment itself, starting with the research concept, presenting the sensors used, the areas and scenarios involved, as well as the processing techniques and metrics used for validation (Section 3.5).

### 3.1. Problem Formulation

The fusion problem in this research can be formulated as combining the spatial positions of camera frames and the laser point cloud. Since LiDAR positioning and measurements are more accurate, it is reasonable to adjust (shift) camera images to specific places in the LiDAR point cloud. This adjustment will allow for the overlay of the camera image over the point cloud, providing joint 3D model with identification possibilities. Thus, the process is similar to the georeferencing of raster data in geographic coordinate systems, though other panes are used as original and reflected surfaces. Nevertheless, the approach based on the adjustment or matching points, known from the georeferencing process, can be used, and as such, it is proposed in this paper. Following this thought, data fusion in this approach becomes a coordinate transformation problem from the local (dynamic) camera frame coordinate system to the fixed world-related LiDAR point cloud coordinate system. It has to be emphasized that, due to the vessel’s movement and the lack of measurement system calibration and integration with other sensors, the camera coordinate system is dynamically changing; therefore, for each and every frame, it is, in fact, a new local coordinate system. The transformation is, in fact, performed from an unknown local system toa known world-related system. Therefore, as the projection for the camera system is unknown, no direct formulas for transformation can be found, and the transformation itself has to be performed based on matching points derived from both measurement sets (LiDAR and camera). The coordinates from the camera (and the frame as a whole) are adjusted (linked) to the point cloud coordinates. The adjustment itself will be performed with the use of neural networks, and the results will be compared to known and used in georeferencing numerical methods. This approach is described in detail below.

### 3.2. Adjustment Points Approach

The adjustment or matching points is known as a method for coordinate transformation in cartography and geoinformatics applications [75,76]. It is also a commonly used and implemented method for data spatial adjustment, as well as for georeferencing in leading GIS software, e.g ESRI ArcGIS (v. 10.8.2). The process consists of two major parts. First, matching points themselves have to be extracted from measurement sets. Second, they have to be somehow adjusted with a computational algorithm. The first task is case-specific and it usually needs an individual approach, tools, and knowledge for research case, while the second task then uses algorithms that are usually implemented in software. In this section, we focus on the first task of points extraction, while in a later section, we describe the adjustment algorithms used in the research.

In the case of camera data acquisition, a few research problems for extracting a set of matching points can be identified and discussed. The first thing to analyze is the effect of camera calibration on the matching points results. Specifically, for a camera with unknown external and internal parameters, determining matching points accurately posed a challenge. Another aspect under investigation was the distance of data acquisition, which involved the planning of measurement profiles. What will the results be if the acquisition is planned 200 m from the shore, and what if it is planned 70 m away? Lastly, however, the most important aspect is how the points will be selected, and whether it is possible to match points that are at the depth of the image when projected onto the plane. All these problems led the authors to prepare multiple test sets for evaluation.

Several datasets, that is, sets with matching points consisting of the coordinates of corresponding points from the LiDAR cloud and the camera image were prepared for the survey (that is, 2 pixel coordinates and 2 LiDAR coordinates in the WGS84 UTM 34N (X, Y) and elevation relative to the water surface (Z).

**Figure 1:**
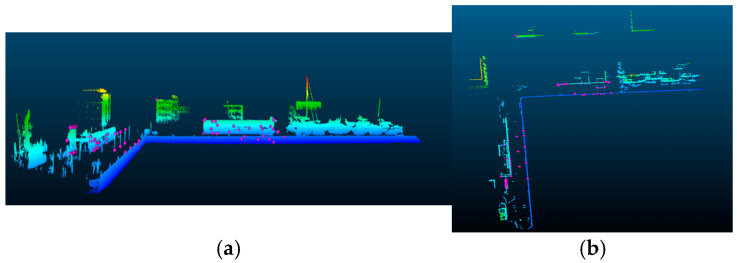
Display of the point cloud in Cloudompare: (**a**) side view and (**b**) top view.

The LiDAR point cloud acquired from the autonomous vessel was used to manually indicate characteristic points located at the shoreline. The CloudCompare program was used for this purpose. It also required cropping the LiDAR point cloud to a range corresponding to the perspective of each individual camera image. In the study, the X coordinate was defined as one of the longitude/latitude coordinates (depending on the perspective of the photo), while the Y coordinate was defined as elevation. Figure 1 shows two different views of the LiDAR point cloud, (a) side view and (b) top view, with selected points highlighted in pink.

The camera dataset was based on pixel coordinates from the images. After marking feature points on the LiDAR point cloud, coordinates were created for the same points on the image. To do this, a Python code was used, whereby clicking on the selected point in the photo recorded the X,Y coordinates of that point. The code used is shown below (Algorithm 1).
**Algorithm 1. Function for obtaining the coordinates in the pixel coordinate system of the photo****Input:** image = cv2.imread(path) im = Image.open(path).convert(‘RGB’)
Camera = ‘image’**def** click_event(event, x, y, flags, params):
  # checking for left mouse clicks
  **if** event == cv2.EVENT_LBUTTONDOWN:
    coordinates = x,y
    x_CAM.append(x)
    y_CAM.append(y*(−1))
    tnp1 = [x,y]
    coordi.append(tnp1)
    r, g, b = im.getpixel(coordinates)
    a = (r, g, b)
    tnp = [coordinates, a]
    result.append(tnp)
    
**if** __name__==“__main__”:
    # displaying the image
    cv2.imshow(‘Camera’, image)
    cv2.setMouseCallback(‘Camera’, click_event)
    # wait for a key to be pressed to exit
    cv2.waitKey(0)
    cv2.destroyAllWindows()**Output:**
Camera coordinates (x,y)

**Figure 2:**
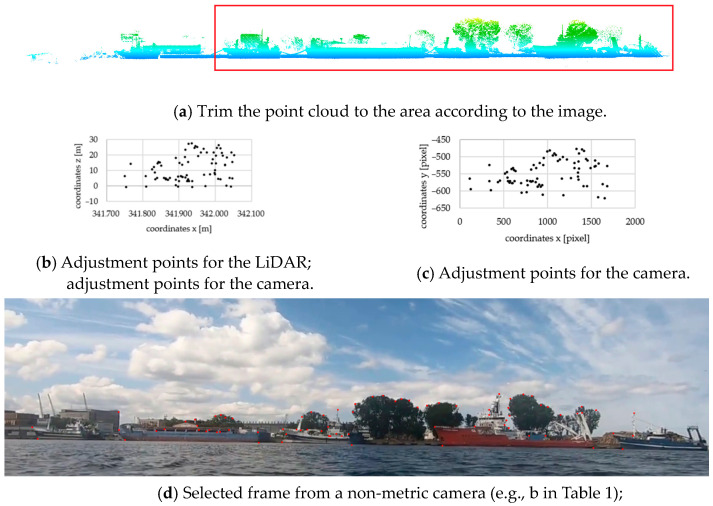
Tests for the expert method with a non-calibrated camera (**a**–**d**).

Figure 2 shows the concept of point matching using one selected photo as an example. In the first step, the LiDAR point cloud was cropped to the area corresponding to the photo (Figure 2a marks the new area with a red rectangle). Next, characteristic points are selected, whose X,Z coordinates are shown in Figure 2b. The projection of LiDAR points onto the plane is always based on the Z coordinate, and depending on the direction of data acquisition, it may correspond to either the X or Y axis. In the presented example, it is the projection of the Z and X points. Figure 2c shows the projection of the coordinates of the pixel points, which are marked in the image shown in Figure 2d. Upon conducting a qualitative assessment, it is possible to see the similarity between both datasets. Quantitative analysis is carried out in the following section.

In the research for this paper, we analyzed 16 examples across 12 different scenarios (the images used in the study are shown in Table 1 in Section 3.5.2). The first 4 scenarios were based on a set from an uncalibrated camera (frames 8, 16, 19, 24). The other images were from a calibrated camera, distinguished by the acquisition distance from the shoreline and the urbanization of the area. For example, in scenarios 2, 3 (frame 8), 8 (frame 116), and 15 (frame 3), it was examined whether normalization or data selection would affect the final results.

### 3.3. Numerical Adjustment Methods

Numerical transformation methods in ArcGIS software (ArcGIS v. 10.8.2). were used to integrate the data. The transformation functions are based on the comparison of the coordinates of source and destination points, also called control points, in special graphic elements called displacement links. For transformations, the from and to locations of the links are used to construct the transformation formulas. By default, ArcGIS software supports three types of transformations: affine, similarity, and projective (desktop.arcgis.com, accessed 30 July 2024). In the study, all three methods were used.

Affine transformation allows for differential scaling, skewing, rotation, and translation of the data. It requires a minimum of three displacement links. Similarity transformation scales, rotates, and translates the data without independently scaling the axes, and does not introduce any skew. A similarity transformation requires a minimum of two displacement links. However, three or more links are needed to produce a root mean square (RMS) error, which is used as a metric. Projective transformation is based on a more complex formula that requires a minimum of four displacement links. The parameters included in the above formula contain information about the internal and external orientation elements of the image. This transformation does not preserve the fidelity of angles or parallelism of the lines, resulting in a change in the shape of the object being imaged [77].

### 3.4. Neural Adjustment Approach

In terms of neural networks, the problem of a coordinate transformation based on point matching can be treated as a regression problem. The goal is to find such a configuration of the network, understood as the network structure and its parameters, which can estimate target coordinates (or the transformation function) with the highest accuracy. The training process is crucial for this task, though the network structure can be also of importance here. Therefore, various structures of the network were analyzed in the research. The nature of neural networks is non-linear, which makes them very useful in many regression and estimation tasks in which traditional numerical algorithms are inaccurate.

It should be mentioned that in this research, being a first approach to using neural networks for this task, traditional network structures were used, and deep AI was not utilized. One of the questions was whether simpler structures are sufficient for this task without the need for using other, more complex ones. Spatial adjustment, for example, in GIS applications, is often performed with numerical methods. Therefore, we propose to verify shallow networks first.

#### 3.4.1. Multiple Layer Perceptron

The multilayer perceptron (MLP) is probably the most popular and commonly used structure of artificial neural networks. It is a classical one-directional network in which the signal is propagated consecutively through all layers from the initial to the final one. The neurons in each layer have no connections between themselves, while they are connected to all neurons from the previous and to the next layers. Each neuron calculates the weighted sum of inputs and processes it with an activation function (typically sigmoidal), calculating the output. The networks can be treated as an input-output model, with connection weights and neuron threshold values as parameters. Such a network, if complicated enough, can model or approximate very complex functions (although not always perfectly). The key aspects are thus the determination of the network structure (number of layers and neurons) and the training process. Many algorithms for this are presented in the literature, among which back-propagation is the most popular [78].

#### 3.4.2. Radial-Basis Function Neural Network

The structure of radial-basis function (RBF) networks does not fundamentally differ from the multiple layer perceptron. The main and major difference, however, is the activation function used for neuron activation. Instead of the sigmoidal function, which is typically used in MLP, RBF activations are radial, being mostly modifications of the Gaussian function. The answer space is thus divided into hyperspheres and not into hyperplanes, as it is in MLPs. In many cases, it can provide advantages over MPLS. Nonlinear functions can be modeled in RBFs using a single hidden layer, and the training of such networks is much faster [79].

#### 3.4.3. General Regression Neural Network

The general regression neural network (GRNN) is a neural implementation of the mathematical general regression algorithm. It is a relatively uncomplicated network with a fixed structure. It always consists of 4 layers: input, hidden (radial), summing, and output. The key role is played by the hidden layer with radial neurons, characterized by the radial transition function. The function center is in the standard that takes account of a given neuron. Each neuron represents one pattern, and each pattern has its own representative; thus, the number of radial neurons is equal to the number of patterns taken into account in the estimation process. The network smoothing coefficient is the parameter controlling the span of the radial networks [80].

In order to use the GRNN network for solving a particular problem, proper quantities should be selected as input and output signals, as well as other network parameters, such as the number of standard units, which is the length of the teaching sequence and the values of the smoothing coefficients of the radial transition functions in standard neurons. In the case of coordinate transformation, the input signal consists coordinates from the original plane, while the output coordinates for the target plane are received.

#### 3.4.4. Linear Networks

Linear networks are another example of the use of network structures for implementing traditional mathematical algorithms. They are used for function approximation with the use of linear models. Instead of the typical matrix convention for algorithm description, in this approach, neural network implementation is proposed. This is, in fact, a two-layer model with linear networks, and the final output is a combination of weights, threshold values, and linear activation functions [79]. In this research, linear neural networks are used solely for the purpose of comparison with numerical methods, as some of them are also based on linear assumptions.

### 3.5. Research Concept

The main objective of the research was to evaluate how artificial neural networks would perform in matching camera–LiDAR points acquired from a specific environment, such as the shoreline zone. The goal was to find and verify the methods addressing the problem formulated in Section 3.1. Two hypotheses were proposed. The first assumed that the matching points approach known from GIS solutions can be used in camera–LiDAR fusion for data alignment, while the second assumed that the use of neural networks is an effective solution for this purpose.

To verify these hypotheses, the research was proposed, including the following tasks. Firstly, the measurements themselves were performed with the use of an unmanned surface vehicle and suitable sensors. Secondly, the datasets consisting of matching points for both measurement sets were created manually and with scripts for various scenarios. The datasets were then processed with neural networks, according to machine learning methodology. Residual errors were calculated. Simultaneously, processing with numerical methods in geoinformatics software was performed. Results from numerical coordinate transformation methods were adopted for comparison with the neural networks. Such a comprehensive approach, with tests in different scenarios, allowed us to see under which conditions the networks adapt better and to verify the hypotheses.

#### 3.5.1. Data Sources and Test Areas

**Figure 3:**
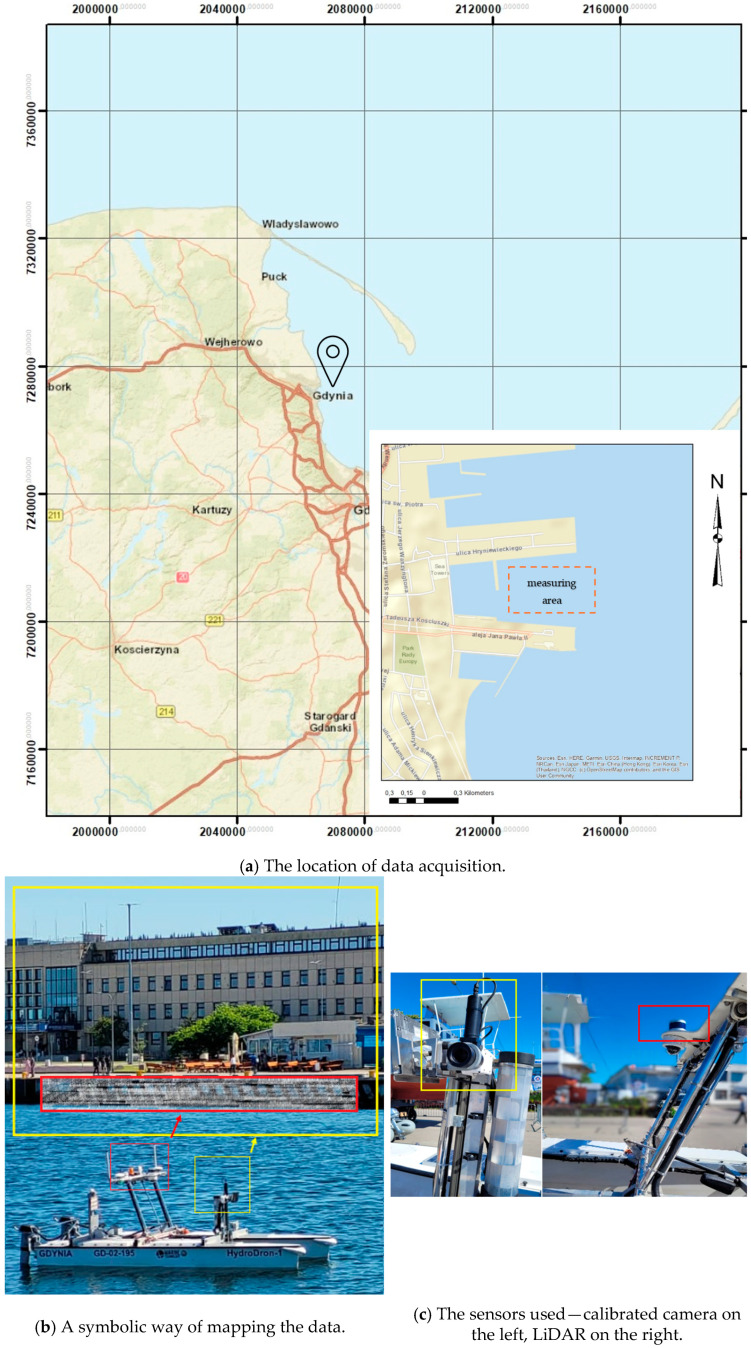
Measurements location and system: (**a**) the measurement area, which is the city of Gdynia, the seaport docks; (**b**) the mapping of data by the autonomous HydroDron-1 vessel. The red part shows the data collected by the LiDAR vessel installed, and yellow color presents the camera’s range of view; (**c**) close-up view of the sensors used, with the lens mounted on the left and the location of the LiDAR on the right.

The measurement site was located in the port of Gdynia, Poland. Data were collected from the Pomorskie Wharf and the Yacht Park, using a Hydrodron-1 (https://marinetechnology.pl/en/hydrodron-2/ accessed on 13 October 2024) equipped with a Velodyne VLP-16 LiDAR. For the first six examples, a GoPro Hero 6 Black camera was used, followed by measurements with a Blackfly S GiGE camera. Figure 3a shows the precise location of the measurements and the data acquisition equipment used. Figure 3b symbolically shows how the information is integrated, where red is the LiDAR and the mapped coastal area, while yellow is the camera mounted on the vessel along with the perspective of the photo that will be taken during the survey. Figure 3c depicts the location where the LiDAR and calibrated camera were placed.

The HydroDron-1 unit, as previously mentioned, is equipped with a camera and LiDAR, as well as two positioning, navigation, and timing (PNT) systems:

- SBG Navsight Ekinox system, which provides angular deviation data (MRU, Motion Reference Unit) and positional data (RTK GNSS);
- Garmin GPS 18x LVC system—a GNSS receiver.

The LiDAR is synchronized with the GPS time scale based on NMEA messages (GPRMC or GPGGA) and PPS signals transmitted by the GARMIN GPS 18x receiver (manufactured by Garmin Ltd, New Taipei, Taiwan). HYPACK 2022 software is used for data acquisition. The camera is connected to the navigation computer operating on Ubuntu 18.04. Data acquisition from the camera is performed using SpinView SDK software (v. 4.1.0.172). Each image captured by the camera contains timestamp information accurate to a single millisecond. The time reference for the camera is obtained from the real-time clock of the Ubuntu 18.04 operating system. The computer’s time is synchronized automatically via internet servers. This procedure allows for precise temporal positioning of each captured image and enables data correlation between sensors.

**Figure 4:**
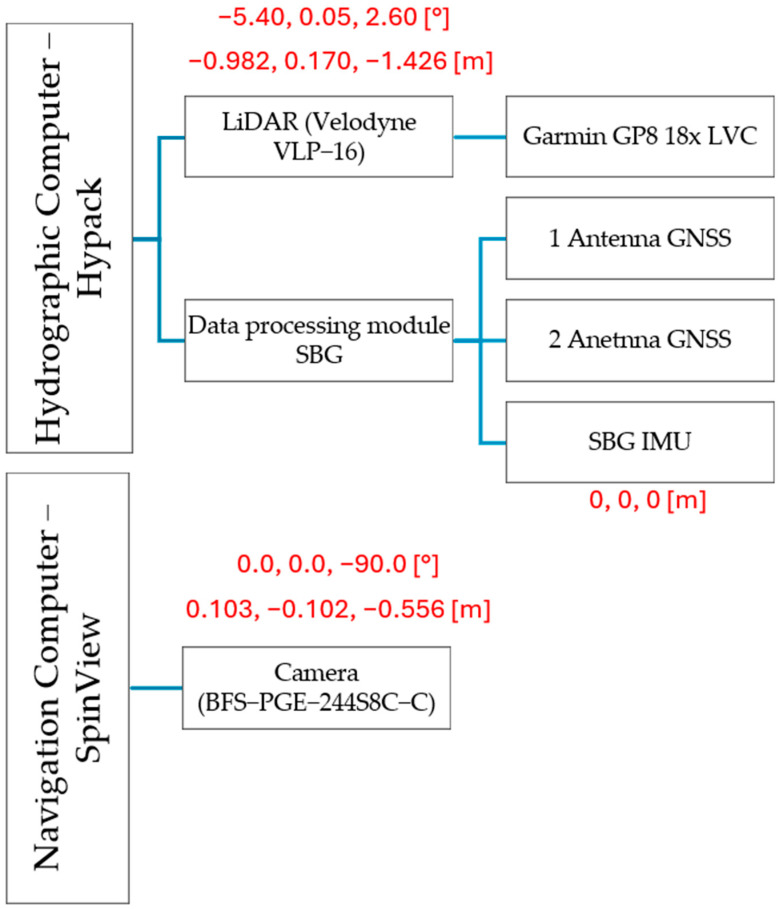
Diagram of time and position synchronization for data acquisition sensors.

The reference position of each sensor is determined relative to the “base_link” coordinate system. The zero point of the base_link system is taken as the zero point of the SBG Navsight Ekinox reference frame. This point is located at the top of the Ekinox IMU housing. The axes of the coordinate system are configured as follows: the *X*-axis toward the starboard, the *Y*-axis in the direction of the vessel’s movement (forward), and the *Z*-axis downward. Figure 4 presents a synchronization diagram of time and position for the data acquisition sensors, with the offsets X, Y, Z [m] and Pitch, Roll, Yaw [°] indicated in parentheses.

The camera calibration was based on photographing a calibration pattern, which is a flat object with known shapes and dimensions. Chessboard fields were used for this purpose, with each field being a square with a side length of 27 mm. The pattern was printed on an A4 sheet. It was photographed multiple times from different angles and camera positions.

For the LiDAR calibration process, a measurement of the Dreamer monument in Gdynia was conducted. This is a slender and static object, with no other objects nearby, allowing for the registration of the monument from both the starboard and port sides. As part of the LiDAR calibration, a calibration measurement was conducted, followed by subsequent procedures such as measuring translational offsets, verifying time synchronization accuracy, and determining angular offsets.

#### 3.5.2. Datasets

Twelve different scenarios were adopted for the study, with selected images shown in Table 1 while and a comprehensive summary with description is shown in Table 2. The first 4 frames (frames 8, 16, 19, 24) were captured with an uncalibrated camera, so the parameters of the internal and external orientation of the photo are unknown. The subsequent images were acquired from a calibrated camera, which also stood out for its better image quality and resolution. The selected frames show a varied shoreline, the acquired images are from different perspectives and a different distance from the shoreline.

**Table 1:** Presents selected images for testing.

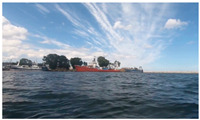	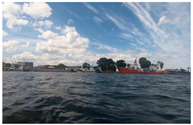	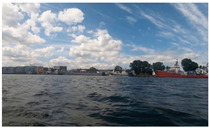
(a) frame 8;	(b) frame 16;	(c) frame 19;
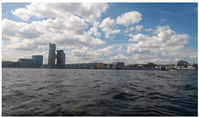	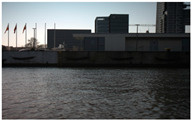	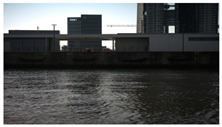
(d) frame 24;	(e) frame 116;	(f) frame 197;
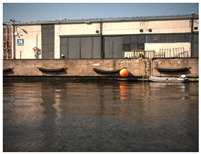	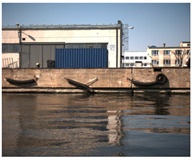	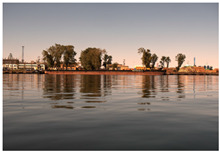
(g) frame 262;	(h) frame 299;	(i) frame 1;
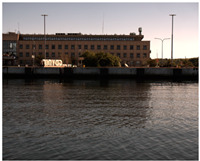	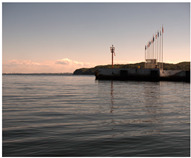	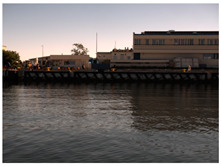
(j) frame 2;	(k) frame 3;	(l) frame 4;

The scenarios are presented below in Table 2. It indicates in which frames an uncalibrated camera was used, as well as how many points in the set were accepted for analysis. The distance of the camera (at the time the image was collected) from the shoreline is then given, and the dominant shoreline type (whether there is more vegetation or urbanized objects) is indicated.

#### 3.5.3. Research Software

The camera data were prepared using the Python language in the Spyder environment. Spyder is a free and open-source scientific environment written in Python for Python, specifically tailored for scientists, engineers, and data analysts.

LiDAR data, on the other hand, were processed using Hypack 2022 and CloudCompare software (v. 2.6.0). LiDAR calibration was conducted subsequent to the measurements at the wharf. The results were then processed and imported into Hypack 2022 software. The next step was to clean and filter the clouds of false reflections and interference. The prepared cloud was then used to establish the matching points. Subsequently, the adjustment of points in two separate coordinate systems was made in ArcGIS software using the Spatial Adjustment tool.

Neural networks were modeled and processed using Statistica Neural Network software v. 4.0. The network structures were selected from the list offered in this module. Coordinates from the camera were used as input values for the network, while LiDAR coordinates were used as output. Thus, each training sample contained 4 values—2 for input and 2 for output. The networks were then created with the use of automatic designer; however, the training process was assisted by the authors. The division of datasets into training, validation, and tests set was performed randomly in this software. The software proposed a set of the best networks for each type, and the authors selected the best one based on the analysis of statistics as well as values for selected single samples. Additional training was guided manually if needed (mostly for GRNN).

The processing of the results and the calculation of residuals and errors were performed in Microsoft Excel (v.2409) with the use of Visual Basic for Applications.

#### 3.5.4. Evaluation Metrics

The assessment of the performance of adjustment methods was based on the residuals in the LiDAR coordinate system. The residuals were the distances between estimated coordinates of matching points from the camera frame after transformation to the LiDAR coordinate system and the same points directly in the LiDAR point cloud. Thus, for each matching point, the residual was achieved, being the Euclidean distance between the coordinates after transformation and the actual LiDAR coordinates. LiDAR, as a geometrically more accurate sensor, was performing the role of ground truth for transformation accuracy analysis.

To indicate a common value for the entire frame, the root mean square error (RMSE) was calculated for the points in each frame. Depending on the needs of the research, the RMSE was calculated for various subsets (training, validation, train), but always for the matching points in a single frame.

The RMSE is a commonly used metric in geoinformatics for assessing any distance-related analysis, as it can reflect both the precision and the dispersion of data. RMSE is calculated based on the Euclidean distance between the coordinates of the *i*-th matching point from the camera after transformation and the coordinates of matched point in the LiDAR point cloud in relation to the number of matching points in the dataset (or subset).

The research results were also assessed quantitatively by the authors in respect to the visual reception of the object in the camera image compared to the achieved results.

## 4. Results

The results of the research are presented in this section in the following order: First, an example of point cloud data and a camera frame is provided, supported by an image fused over the point cloud. This shows the general concept of the process and gives an overview for assessment of numbers. In the next sections results for numerical and neural methods are provided. Then, a comparison of the neural and numerical research is presented, followed by the discussion of the results.

### 4.1. Data and Fusion Examples

**Figure 5:**
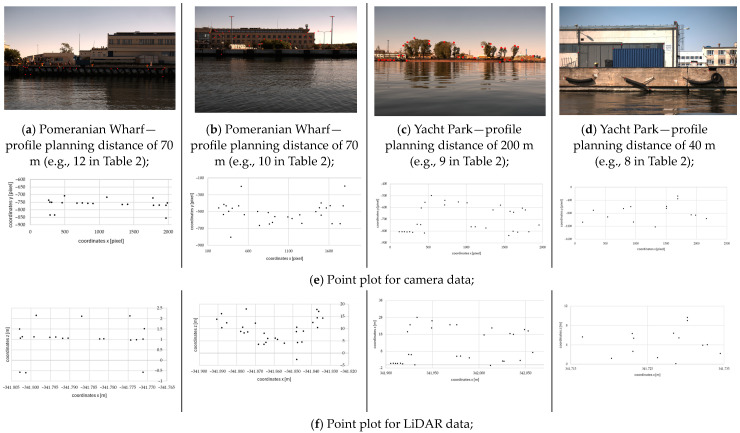

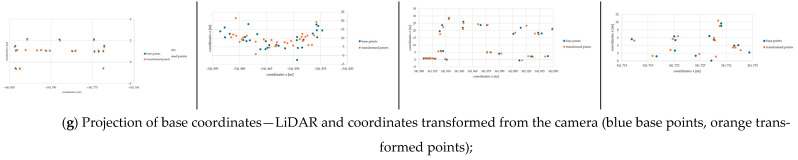
The figure shows, in parts (**a**–**d**), the frames from the camera with the selected points; in part (**e**) the coordinates of the points from the camera; in part (**f**), a plot of the identical points extracted from the LiDAR point cloud; in part (**g**), projection of base coordinates—LiDAR and coordinates transformed from the camera.

The datasets were tested under twelve different scenarios according to the concept. The planned distance of the profile from the shore was in the range of 40–200 m. The feature points selected for analysis were based on objects located at various distances from the shore, near the shore (signs, ships, quay), and inland (walls of buildings, windows, pillars). Figure 5 presents a few selected frames, where red points indicate the selected characteristic points for matching:

(a) Measurement sets for acquisition from 70 m from the shore, points selected in a linear manner, characteristic objects on the shore;
(b) Measurement sets for acquisition from 70 m from the shore, points selected in a scattered manner, characteristic objects at a considerable distance from the shore;
(c) Measurement sets for acquisition from 200 m, due to the wide perspective of the image, points selected in both a linear and scattered manner;
(d) Measurement sets for acquisition from 40 m, points selected in a manner both on shore and inland.

Figure 5e shows the result of projecting pixel coordinates, Figure 5f shows LiDAR coordinates, and in Figure 5g, using numerical transformation methods, a comparison of the LiDAR coordinates with the transformed camera points is presented. After qualitative analysis, the best set of measurements was used to match the image to the LiDAR point cloud.

**Figure 6:**
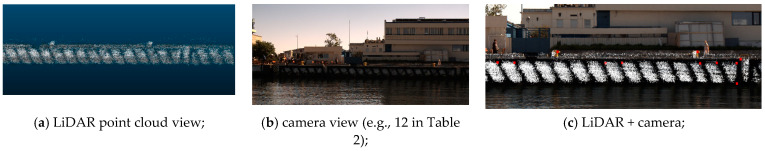
Integration of camera and LiDAR data (**a**–**c**).

Figure 6 below shows the data fusion process. Using the matching points, the camera image was fused into the entire LiDAR point cloud. Numerical methods were deployed for this point matching.

### 4.2. Numerical Transformation

Based on the research concept given in Section 3.5, all three spatial matching methods available in ArcGIS were tested: affine, projective, and similarity. The results are presented in Table 3. The RMS error was calculated in ArcGIS using the Spatial Adjustment tool.

Table 3 shows that the results vary widely across the examples. The smallest errors are even less than 0.2 m, but on the other hand, large errors, such as 7 m, appear. This shows that the results of the transformation depend largely on the parameters of the equipment and the method by which the points are selected.

The best results were obtained using the projective method. Numerical methods do not perform well when performing transformations on an uncalibrated camera. This may be due to the fact that due to the inferior resolution of the image, it is difficult to unambiguously indicate a point in the image that is identical to a point from the LiDAR cloud. In the case of images from a calibrated camera, significantly better results were obtained. The exception is example 13; this may be due to the fact that the points indicated in the photo were based on elements located far from the edge. The perspective of the photo and its depth did not allow us to find the correct relations used for the transformation parameters.

The analysis shows that numerical methods on prepared sets of points, based on the identification of homologous points on the shore itself, at the docks, work best. Using numerical methods, we were able to obtain, in a few cases, error values within 12 cm, which is a very good result compared to some examples where errors are within a few meters.

Another important observation is that the number of points adopted for analysis is not very important. Under theoretical assumptions, one should prepare a set of only three homologous points to perform the transformation. The more of these points there are, the more accurate the results will be. However, the average error can only yield good results from a local perspective. This means that the farther one is away from the analyzed points, the worse the matching will be. Therefore, it is not recommended to select datasets by eliminating points with the largest errors.

### 4.3. Neural Transformation

All scenarios have also been tested with artificial neural networks. The goal of this part was to check and compare the performance of various networks for this task, namely radial basis networks, multiple layer perceptron, and general regression neural networks.

For training and verifying the networks, an approach known from machine learning was used. The dataset was divided into three parts: the training set, used directly for teaching algorithm; the validation set, which is data used within the teaching process as an external verification, not used directly for teaching; and the test set, containing data not used for teaching but to calculate the error of the already-taught network. Dataset division was established randomly by the software. Generally, the proportion used was roughly 70% for training, 20% for testing, and 10% for validation. In a few cases, this caused a problem of a very small validation set, but we assumed that it is important to use as many cases as possible for direct training.

In Table, four RMS errors for the analyzed networks are given. The results are presented separately for the training, validation, and test datasets. The numbering of the examples is the same as in Section 4.2. The division given in the table reflects the different cameras and distances used in the scenarios.

Table 4 shows that the results vary significantly among the examples. The smallest errors for the test set are even less than 0.4 m, but on the other hand, large errors like 25 m appear. This shows that the results of the transformation rely very much on the parameters of the equipment and on environmental issues, such as measurement distance, which directly affect the accuracy of matching points.

Generally, the values of errors for the test sets confirm the values achieved for the training set; however, in some cases, they are significantly worse (e.g., GRNN in example 4), which illustrates the effect of overfitting (overteaching) to the training set. The most varied results were achieved for that validation set, which is reasonable, as this was always the smallest set. In general, it should be noted that in a few scenarios, very small datasets were used. There was a risk that the set would be too small to teach the networks. It is somewhat reflected in the third row (example 1 with selected number of points), where the results are worse than with the full set for this frame. However, the results for examples 4–7, which had very small teaching sets, show that other factors, such as distance to the shore and calibration, are much more important.

Generally speaking, the best results were achieved with the use of RBF, while the worst was with MPL; however, this will be analyzed in more detail later.

**Figure 7:**
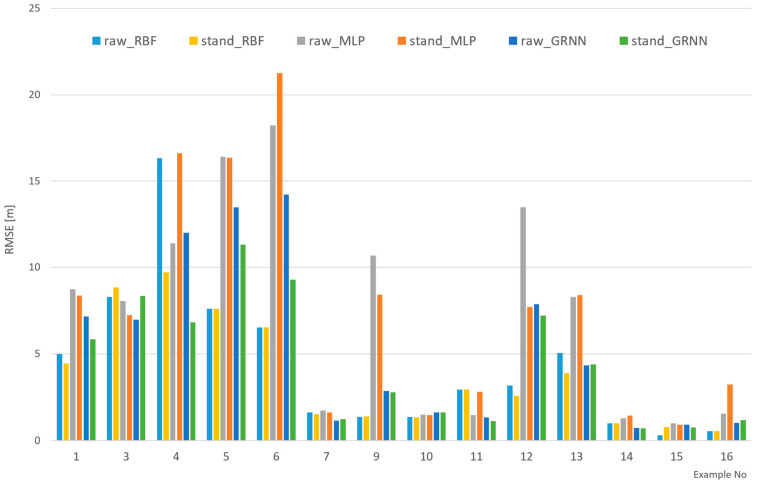
RMSE for non-standardized (raw) and standardized datasets.

While analyzing the results, it was noticed that the errors for the vertical component was generally smaller than that for the horizontal component, which is not reflected directly in the joint RMS for position. It is assumed that this may be related to the nature of coordinates for LiDAR in the UTM system, where the horizontal coordinate is much larger (six digits—millions of meters) than that of the vertical (two digits—tens of meters). Therefore, it was decided to standardize all the values in the datasets prior to the teaching process. The results were ultimately recalculated back to the coordinate system to calculate the errors. Figure 7 shows the results for this part of the analysis. To compare them jointly and to avoid an unreadable figure, the joint RMS for all sets is given (train, validation, and test); therefore, the RMS for entire datasets are presented with and without standardization for various networks. Example 2 and example 8 are omitted as they are a standardized version of example 1 and example 7.

Figure 7 shows that, in general, the standardization of datasets has a positive impact on the results. RMSE, in most cases, is smaller. The difference, however, is not very significant. A probable reason for the small difference is the pre-processing, which is automatically done in Statistica Neural Networks software while performing teaching. This process can involve various scaling techniques. It can be seen in the figure that the most sensitive to standardization is MLP; however, in some examples (2, 3), GRNN also shows significant differences, and in example 3, with a selected number of points, RBF also shows significant differences.

Considering the above analysis, the results for the sets after standardization will be shown in the following results.

**Figure 8:**
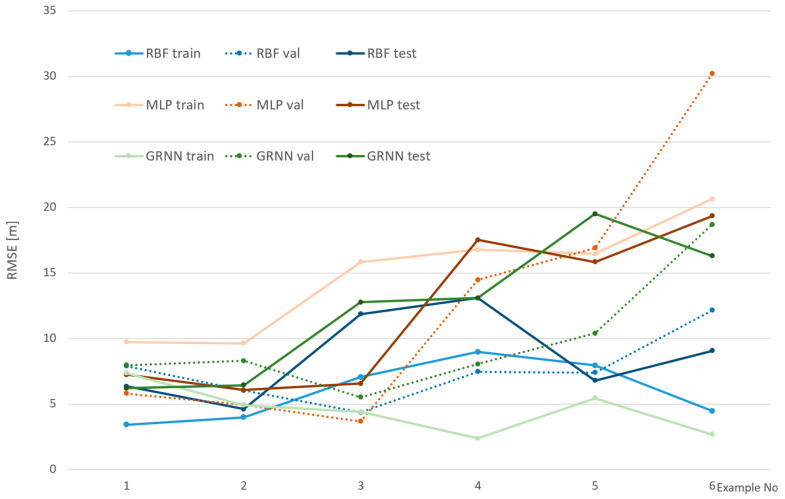
RMSE for various neural networks for examples 1–6.

**Figure 9:**
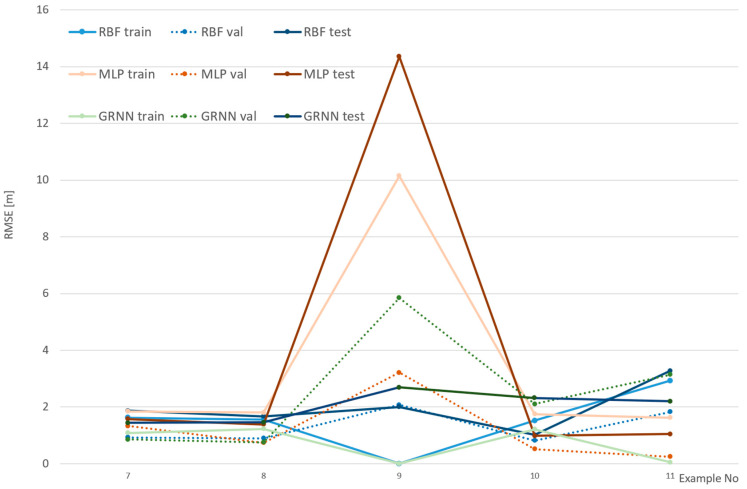
RMSE for various neural networks for examples 7–11.

**Figure 10:**
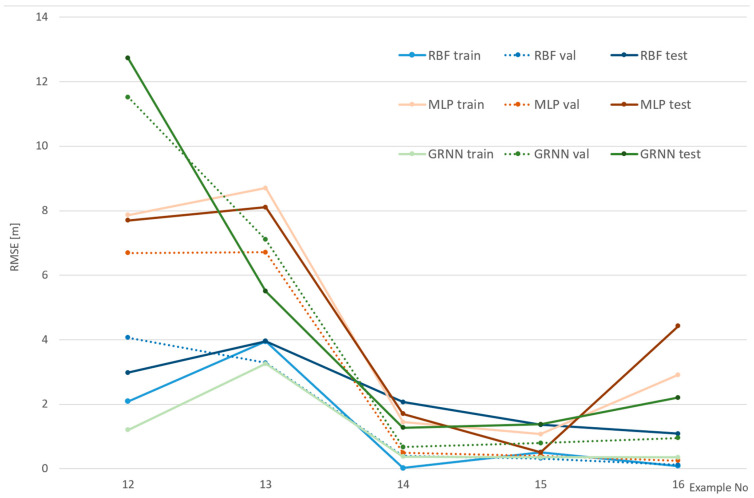
RMSE for various neural networks for examples 12–16.

Figure 8, Figure 9 and Figure 10 show the efficiency of the analyzed networks, reflected in RMSE, which was the basic goal of this part of the research. The examples were divided into three figures to make them clearer and to reflect various cameras and environments. It should be noted that the scale on the vertical axis is different in each figure, as it reflects the values.

In Figure 8, RMS errors for examples 1–6 are shown. In these cases, a non-calibrated camera was used at a distance of 70 m. RMS errors for various sets and networks are presented. It can be observed that, generally, MLP gives the worst results. Only in one case does the GRNN test set have a larger RMS than MLP. GRNN generally seems to be a bit overfitted in most cases, as it performed the best for the teaching set, but not so well for the others. However, this is always a compromise regarding error distribution between sets. Looking at the shape of the line, it can be observed that GRNN is generally not sensitive to validation, which corresponds with the structure and teaching concept of this network. The worst results were achieved for example 6, which might suggest that smaller sets can negatively influence network efficiency. This is also confirmed in the case of test sets for MLP and GRNN in examples 1 and in example 3. The best balance between sets was achieved in the case of RBF, and this can be chosen as generally the best network in this part of the research.

In Figure 9 RMS errors in examples 7–11 are shown. In these cases, a calibrated camera was used at a short distance (40 m). This caused problems with finding and distributing matching point, so the set was relatively small. The results show, however, that it was not crucial for the accuracy of the transformation.

The results shown in Figure 9 generally confirm earlier findings related to the comparison of the networks. Again, MLP gives the worst results, and again, GRNN fits the best to the training set. This time, however, GRNN performs better on the test set, which might be the result of point distributions in a smaller area. Generally, the results achieved are much better than those from Figure 8, which shows that the kind of camera and the distance to shore are crucial for fitting accuracy.

To further investigate these influences, Figure 10 shows the results for examples 12–16. This time, a calibrated camera is also used, but the distances are larger—200 m for example 9 and 70 m for the others.

In Figure 10, the effect of the large distance is clearly visible in example 12 for the GRNN network. This time, overfitting (best values for the training set) led to major errors (even larger than MLP) for the test and validation sets. Another interesting case is example 13, in which the effect of problems with localizing matching points in the photo arises. This was described in Section 4.2 for numerical results, and it can also be noticed here. Again, the best results were achieved with the RBF network; however, it should be noted that for examples 14 and 15 MLP was more accurate. Interestingly MLP achieved better results for the test set than the training set, which shows that it is the specificity of these particular points.

In summary, for part of research, it can be said that neural networks can be used as approximators for coordinate transformation with point matching algorithms in this type of fusion. The best results were achieved with the RBF network, while the worst was with the MLP. The influence of camera calibration is crucial, while the influence of distance is major. The problem of overfitting during the training process may appear, as seen especially for the GRNN network.

### 4.4. Comparison of Neural and Numerical Methods

In this section, a part of the research focused on the verification of neural methods against numerical methods is presented. To make this possible, a modification in the research methodology had to be proposed. In the first approach, we tried to copy the approach from numerical methods (as given in Section 4.2) to neural methods; namely, we used all samples from the dataset as the training set. This resulted in the perfect adjustment of the networks to the teaching values. The achieved RMS was each time equal to 0—no errors were made, and a perfect fit was established. Typical overfitting occurred, as the networks made mistakes for any additional point.

Taking this into consideration, the second approach to the research was proposed, which could be conducted with both neural and numerical methods. The idea was to maintain a test set for all methods while using the entire validation set as the training set. As a result, each dataset was divided into a training set and a test set. For the numerical methods, the adjustment links were established only for the training sets, while RMS errors were calculated for both the training and test sets. For the neural methods, no validation was used in the training algorithms, and all data from this set were used for teaching. RMS errors were then calculated separately for the training and test sets. Thus, in both approaches, common training and test sets were used, allowing a direct comparison of the methods. However, it should be remembered that it is different from the classical approaches given in Section 4.2 and Section 4.3. Generally, the results in these sections are worse than those in the previous ones (especially for neural networks).

The results are presented in Table 5. It should be noted that the methods giving the worst results—MPL for neural networks and Similarity for numerical methods—were omitted from this table to make it more readable. However, a new kind of network was introduced: linear. This is a very specific network without any non-linear elements inside its structure. In many cases, it can, however, give surprisingly good results, reducing the computational burden of traditional networks. It was included because numerical methods are often based on linear assumption, so it might be interested to compare them.

**Figure 11:**
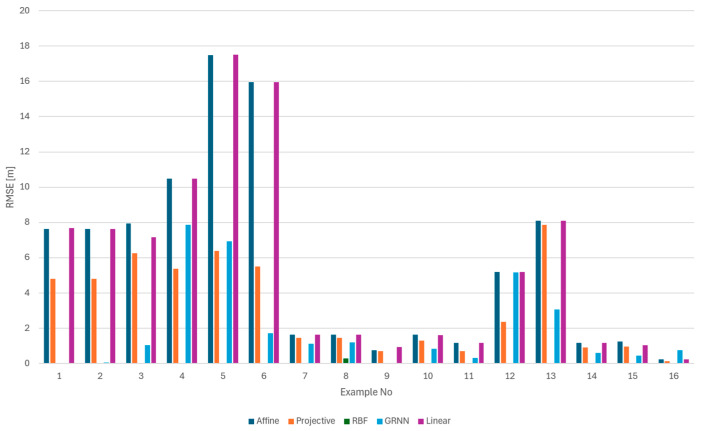
RMSE for selected methods for the training set in comparative research.

**Figure 12:**
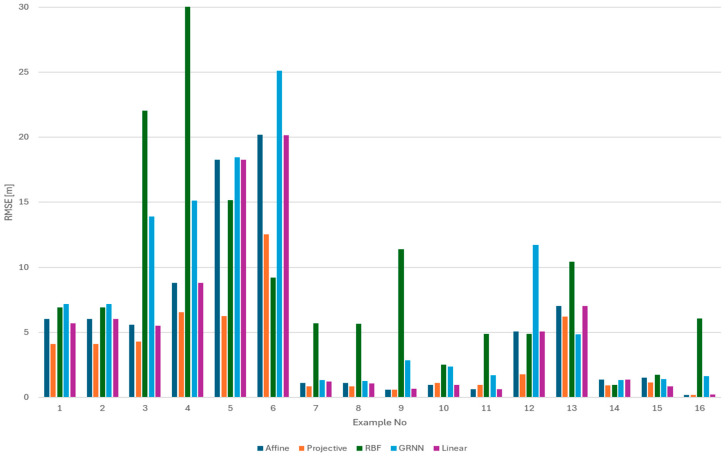
RMSE for selected methods for the test set in comparative research.

The analysis was performed first separately for the training set (Figure 11) and for the test set (Figure 12). The values for each method are presented individually. The results obtained for both the numerical and neural groups of methods can show their dependency on the scenarios used. For both the learning set and the test set, one can see a significant disparity in the results depending on the equipment used. Camera calibration has a very strong influence on the results.

**Figure 13:**
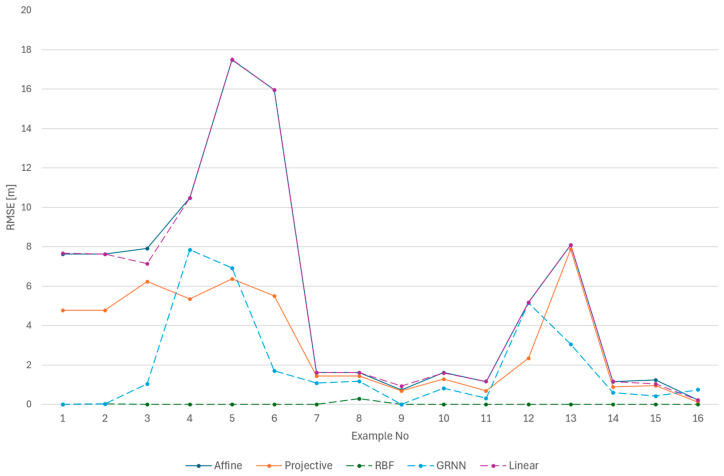
RMSE for selected methods for the train set in comparative research.

The analysis of the table and Figure 11 provides interesting observations about models’ responses to the training set. In fact, it represents the level of fit to the data, as no validation was used. First of all, it can be noted that the values vary greatly between the examples—ranging from very small errors less than 0,2 m, up to 8 m, with significantly worse results for examples 12 and 13. This is the result of the long measurement distance to the object. Example 13 is particularly interesting; the distance of the profile to the shore was 70 m; however, the observed object was significantly farther. This eventually resulted in numbers similar to those from example 12 in which the shore was at a distance of 200 m. Secondly, it can be observed that the camera type influences the results even more. While, for a calibrated camera, the RMSE is generally smaller than 2 m (for distances up to 70 m), for a non-calibrated camera, it is usually larger than 5 m, especially in the case of numerical methods and linear networks (which, in fact, is a network structure for the numerical method). Very interesting observations can be made regarding RBF networks. In almost every example, perfect fitting to the training set was observed, resulting in no RMS error. As can be seen in Figure 13, such great performance was not confirmed for the test set. This means that removing the validation set caused major overtraining for the RBF network. The same effect, although at smaller scale, can be observed for the GRNN network. This, however, was not observed for the MLP network (which is not included in the graph).

Prior to the analysis of Figure 12, it should be noted that the RMS for the RBF network in example 4 exceeds the scale (more than 60), but the scale was adjusted to make other values visible. Figure 11 generally confirms earlier observations; however, this time, large errors can be seen for the RBF and the GRNN (for most cases nearly twice as larger than for the other methods). This clearly shows that validation is crucial for the training of the neural networks. It is clearly visible when comparing these results to the ones obtained in Section 4.3, where networks trained with validation sets were analyzed. A new aspect of this part of the research was the introduction of the linear network. Figure 11 and Figure 12 show that, in most cases, its results are nearly the same as those for the affine numerical method.

To compare the results for the training set and the test set directly, Figure 13 has been prepared. It contains a linear graph with all the values from Table 5. Again, the scale of the vertical axis is reduced to show most of the values, leaving the RBF for example 4 outside of the scale. The graph for the affine method is, in many parts, covered by the graph for the linear network.

The analysis of Table 5 and Figure 13 confirms previous findings about the major influence of scenario parameters. Additionally, significant differences can be observed in the results for the training and test datasets in the case of neural networks. This leads to the conclusion that, in this part of the research, numerical methods were much more stable. The main reason for this is the poor training process for the networks due to a lack of validation sets.

**Figure 14:**
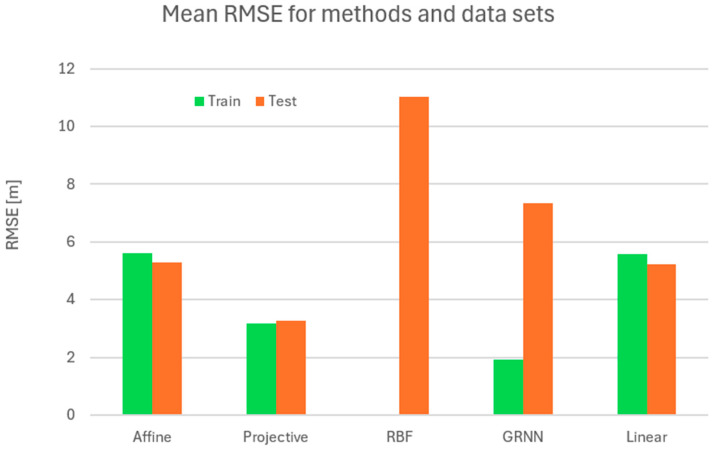
Mean RMSE for selected methods for training and test the test sets.

**Figure 15:**
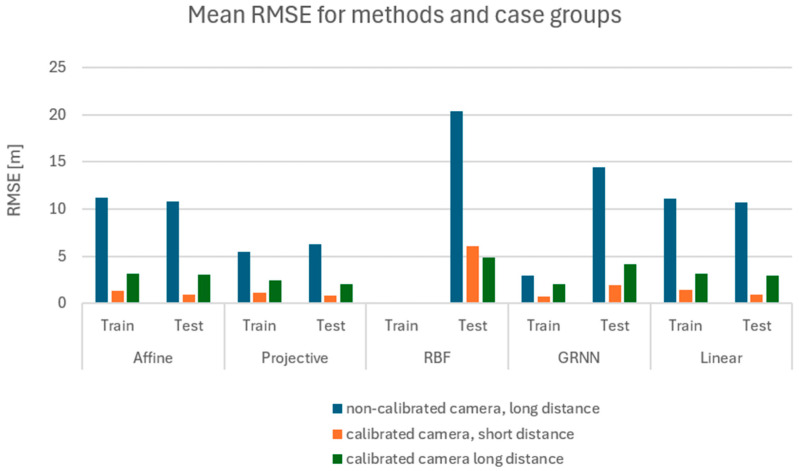
Mean RMSE for selected methods for training and test sets in case groups—non-calibrated camera, long distance (cases 1–6); calibrated camera, short distance (cases 7–11); calibrated camera long distances (cases 12–16).

**Figure 16:**
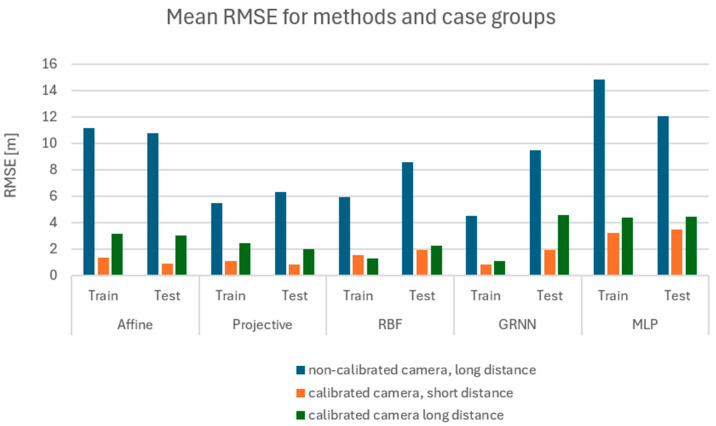
Mean RMSE for selected methods for training and test sets in case groups: non-calibrated camera, long distance (cases 1–6); calibrated camera, short distance (cases 7–11); calibrated camera long distances (cases 12–16)—validation used during training.

To make the analysis more comprehensive, additional statistics are presented in Figure 14, Figure 15 and Figure 16, namely the mean, maximum, and minimum values of RMSE in various configurations.

In Figure 14, the mean RMSE for selected methods is presented for the training and testing datasets. The overfitting of RBF and GRNN can be clearly observed, as indicated by the zero or near-zero RMSE for the training set and significantly higher RMSE for the testing set. This suggests that the models might require better regularization or a larger dataset to improve generalization. On the other hand, these networks can fit nearly perfectly to known points. In general, the projective method achieved relatively low RMSE values for both the training and testing sets, indicating model stability. The analysis of the minimum and maximum values also confirms these findings. The RBF network, in particular, seems to be very unstable, as it has the smallest minimum value and the largest maximum value.

To analyze the influence of environmental and camera settings on the efficiency of the algorithms, Figure 15 has been prepared. It shows the RMSE for various methods grouped by scenarios. Each algorithm performed best with the calibrated camera and short distances. It is evident that calibration can significantly reduce errors, even for longer distances. This impact is even greater for the neural methods. The RBF network for the test set is the only case in which the results were better for the calibrated camera at a long distance than at a short distance. This shows the effect of model overfitting. It must be, however, remembered that this comparison thus far was based on results obtained without a validation process during the training of the networks. Implementing this feature can help overcome the overfitting problem. In Figure 16, a graph similar to Figure 15 is presented, but the networks are trained with validation.

Figure 16 shows the comparison of mean RMS for various methods in case groups, as in Figure 15, but validation was used during the training of neural models. Additionally, MLP is given instead of a linear network. The scale on vertical axis is also different, as the values are better. It can be noted that the effect of overfitting is much smaller and, in the case of RBF and MLP, are nearly equal for the training and test sets. Only for GRNN is this effect significant. The results for training and test sets are comparable for the projective and RBF values. RBF and GRNN perform better than numerical methods with a calibrated camera at long distances for the training sets.

The results generally confirm that neural networks can be an alternative to numerical methods only if they are properly trained with a validation set. A comparison of neural and numerical methods should be conducted jointly based on all research from Section 4, as the values in Section 4.4 are not fully representative. The networks can perfectly adjust to the sample points if we present them as training data. However, problems may arise with points outside of the set.

## 5. Discussion

The results of the research presented in Section 4 allow for an analysis of the problems undertaken from various aspects. A major part of the research is Section 4.3, in which the efficiency of neural networks was verified; however, the comparison to numerical methods in Section 4.4 and the analysis of numerical methods themselves in Section 4.2 are also interesting, providing a jointly complex insight into using point matching algorithms in LiDAR–camera fusion.

The research demonstrated that camera calibration significantly impacts image quality and the accuracy of results for both numerical and neural methods. The fusion of information from images acquired using an uncalibrated camera produced the largest errors, which were several times greater than those from a calibrated camera.

Another important factor identified in the observations is the distance to the object from which the points are collected. Camera data should be acquired as close to the edge as possible but far enough to capture the required range of the scene. It is also essential to limit the integration of LiDAR and camera information to objects close to the shore; otherwise, accuracy will be affected, as demonstrated in Example 13. The results indicate that the distance from the shore is a significant factor in the accuracy of matching.

These findings show that the methods and procedures of data acquisition have a substantial influence on the overall accuracy of data fusion. Data processing cannot compensate for issues related to the quality of the input data. In addition to these factors, it is crucial to consider that the selection of matching points can also significantly impact the results. In our examples, this selection was done manually, and since it was based on expert judgment, we assumed it did not influence accuracy. However, in automated systems, this could be an important factor, warranting further research in this area.

A few techniques for pre-processing measurement data were tested. For the numerical approach, standardization, normalization, and selection did not improve the results. In contrast, for the neural approach, each of these treatments improved the final results to some extent; however, the improvements were not significant. One can therefore assume that even better results could be obtained if the test sets were prepared more thoroughly, taking into account the problems encountered later in the processing phase.

Overall, the results confirmed that LiDAR-camera fusion is achievable using a point-matching approach, even without a priori knowledge about vessel movement. The data processing numbers with various methods are highly dependent on the quality of the input data; nonetheless, in many cases, the results were satisfactory enough to enable visual fusion. It seems that matching errors within a few meters can be considered acceptable, while errors of less than 1 m are satisfactory for this type of information fusion. Such results were achieved in many examples, not only with numerical methods but also with neural ones. In some instances, neural networks provided better results than numerical methods. Generally, the best outcomes were achieved with the RBF network; however, in specific cases, the MLP and GRNN also produced good results.

That said, the networks did not perform as well as expected when considering the generalization capability of the model. The networks provided outstanding results for the training set, indicating that if all crucial points are selected, the adjustment can be made perfectly. This again leads to the conclusion that processing algorithms cannot compensate for the accuracy of matching points

## 6. Conclusions

The goal of the presented research was to analyze the possibility of using a point-matching approach for data alignment in radar–camera fusion on USVs and to verify the usability of neural networks for this purpose. The research question was whether using this concept can offset the inaccuracies caused by the lack of full calibration of the system and the lack of full information about vessel movement. A crucial issue in this research was the dynamic environment of the floating platform, namely the unmanned surface vehicle, which is challenging for data processing and makes this research unique.

Through a series of tests in different scenarios, the dataset selection method for the camera images was designed so that both numerical and neural methods could be used for spatial matching. The dataset from various scenarios was then processed with different neural and numerical methods.

The research has positively verified the first hypothesis: The point-matching approach can be used for LiDAR–camera fusion. The second hypothesis, regarding neural networks being efficient tools for this, was proven partially. While they can be employed for this task and radial basis function networks were found to be the most effective for this, the results are, however, highly dependent on factors beyond the processing method itself. The networks can provide matching without using the information about platform movement from other sensors, but they cannot overcome the inaccuracies of matching points themselves. Therefore, the most important findings of this research are related to identifying the parameters that have a crucial impact on the process.

The first crucial parameter is distance. Characteristic points close to the coastline should be selected for satisfactory matching results, and the profiles should not be far from the shoreline. This is a valuable conclusion and a good direction for further research.

The second crucial parameter is camera specifications. So-called non-metric cameras should not be used for the purpose of shoreline observations with automatic surface vehicles, as they introduce too much error.

The third important factor is the accuracy of selecting the matching points themselves. This is particularly crucial for future research and implementation in automated systems. Selection algorithms should aim to choose points that are close to the shoreline and characteristic enough to be easily identified. This highlights the direction for future research. We plan to explore various approaches for automating the point selection process, including AI algorithms.

In summary, the research demonstrated that neural methods can be used as an alternative to numerical calculations and that the point-matching approach is appropriate. With this knowledge, the neural approach can be leveraged to enhance the computational process. Regarding the achieved accuracy, it should be emphasized that camera imagery usually provides some assistance in coastal analyses; therefore, accurate metric analyses are conducted on point cloud LiDAR data. Image data can provide additional information for the identification and analysis of certain characteristic objects, leading to the conclusion that the achieved accuracies under specific conditions are satisfactory. It should be emphasized that these results were obtained without prior knowledge of the platform’s dynamics in a water environment.

It should be noted that the proposed approach currently has some limitations that need to be addressed in future research. First of all, platform dynamics should be included in future research to achieve the transformation of local coordinate systems, accounting for position and orientation corrections. Secondly, the automation of the method needs to be improved. The algorithms must be developed to automatically identify matching points using spatiotemporal synchronization or advanced image processing techniques. Finally, a more advanced approach to data fusion can be proposed, focusing on features rather than only points. For these purposes, advanced AI methods will be employed to detect characteristic points in both the images and the point clouds. It is also planned to use a convolutional network for object detection and segmentation based on LiDAR point clouds and RGB images.

In general, further research directions will concentrate on developing a method that enables spatial-temporal synchronization of LiDAR and camera data using AI for more sophisticated and automated data processing.

## Figures and Tables

**Table 2:** Description of the scenarios tested.

Example	Calibrated Camera? [Yes/No]	Number of Match Points	Distance from Shore [m]	Predominant Type of Shoreline [Urbanised/Vegetated]
1 (frame 8)	no	56	70	vegetated
2 (frame 16)	no	79	70	vegetated
3 (frame 19)	no	65	70	vegetated/urbanized
4 (frame 24)	no	39	70	urbanized
5 (frame 116)	yes	27	40	urbanized
6 (frame 197)	yes	11	40	urbanized
7 (frame 262)	yes	15	40	urbanized
8 (frame 299)	yes	14	40	urbanized
9 (frame 1)	yes	30	200	vegetated
10 (frame 2)	yes	30	70	urbanized
11 (frame 3)	yes	31	70	urbanized
12 (frame 4)	yes	21	70	urbanized

**Table 3:** RMS errors using numerical methods.

Example	Scenarios	Calibrated Camera? [Yes/No]	Number of Match Points	Distance from Shore [m]	RMSE [m]		
					Affine	Projective	Similarity
1	frame 8	no	56	70	7.22	4.61	8.10
2	frame 8	no	56—rescaled	70	7.22	4.61	8.10
3	frame 8	no	11—selected	70	2.34	0.83	4.03
4	frame 16	no	79	70	10.16	5.56	10.44
5	frame 19	no	65	70	17.54	6.28	18.24
6	frame 24	no	39	70	16.53	6.01	21.36
7	frame 116	yes	27	40	1.49	1.35	1.52
8	frame 116	yes	11—rescaled	40	1.49	1.35	1.52
9	frame 197	yes	11	40	**0.53**	**0.53**	**0.54**
10	frame 262	yes	15	40	1.50	1.24	1.59
11	frame 299	yes	14	40	**1.08**	**0.72**	**1.40**
12	frame 1	yes	30	200	5.15	2.20	5.25
13	frame 2	yes	30	70	7.80	7.46	8.00
14	frame 3	yes	31	70	1.14	0.87	2.11
15	frame 3	yes	27—selected	70	0.82	0.36	1.51
16	frame 4	yes	21	70	**0.22**	**0.12**	**0.24**

**Table 4:** RMS errors for neural networks in the research.

Example	RBF—RMSE [m]			MLP—RMSE [m]			GRNN—RMSE [m]		
	Train	Validation	Test	Train	Validation	Test	Train	Validation	Test
1	3.41	7.88	6.34	9.72	5.80	7.23	7.36	7.96	6.21
2	3.97	6.01	4.59	9.61	4.91	6.06	4.90	8.29	6.43
3	7.05	4.35	11.86	15.83	3.67	6.55	4.39	5.50	12.76
4	16.44	8.42	12.94	10.81	17.12	8.23	6.90	23.62	14.68
5	7.94	7.40	6.78	16.27	14.67	17.83	7.94	19.09	19.39
6	4.46	12.13	9.07	16.98	10.24	23.95	8.07	15.38	25.89
7	1.62	0.93	1.87	1.85	1.33	1.58	1.08	0.85	1.44
8	1.56	0.90	1.67	1.80	0.74	1.39	1.23	0.75	1.46
9	0.00	2.07	2.00	10.15	3.22	14.36	0.00	5.85	2.69
10	1.52	0.82	1.02	1.75	0.51	0.98	1.21	2.10	2.32
11	2.93	1.83	3.27	1.62	0.25	1.04	0.05	3.14	2.20
12	2.49	5.21	3.77	13.67	8.66	14.63	3.62	12.61	12.67
13	4.79	5.12	5.72	9.12	5.43	6.66	3.16	7.13	5.52
14	0.00	0.39	2.07	1.42	0.60	1.06	0.27	0.73	1.40
15	0.00	0.19	0.65	1.19	0.35	0.37	0.93	0.71	0.90
16	0.08	0.16	1.07	1.40	0.16	2.12	0.69	0.36	1.67

**Table 5:** Summary of results for neural networks and numerical methods.

Example	Numerical Methods—RMSE [m]				Neural Methods—RMSE [m]					
	Affine		Projective		RBF		GRNN		Linear	
	Train	Test	Train	Test	Train	Test	Train	Test	Train	Test
1	7.63	6.02	4.79	4.11	0.00	6.92	0.00	7.17	7.67	5.69
2	7.63	6.02	4.79	4.11	0.03	6.93	0.04	7.17	7.63	6.02
3	7.93	5.59	6.24	4.31	0.00	22.02	1.04	13.89	7.15	5.51
4	10.49	8.82	5.36	6.55	0.00	61.77	7.86	15.14	10.49	8.82
5	17.50	18.26	6.38	6.26	0.00	15.16	6.92	18.44	17.51	18.26
6	15.97	20.19	5.51	12.54	0.00	9.21	1.72	25.11	15.97	20.15
7	1.62	1.12	1.45	0.86	0.00	5.71	1.10	1.32	1.63	1.23
8	1.62	1.12	1.45	0.86	0.29	5.65	1.19	1.28	1.62	1.08
9	0.74	0.61	0.70	0.61	0.00	11.39	0.00	2.84	0.94	0.69
10	1.62	0.95	1.30	1.12	0.00	2.53	0.83	2.38	1.61	0.96
11	1.17	0.65	0.70	0.96	0.00	4.88	0.31	1.71	1.17	0.65
12	5.19	5.08	2.36	1.77	0.00	4.89	5.15	11.74	5.18	5.08
13	8.09	7.02	7.87	6.21	0.00	10.43	3.07	4.85	8.09	7.03
14	1.16	1.37	0.90	0.94	0.00	0.95	0.60	1.33	1.17	1.37
15	1.25	1.51	0.96	1.14	0.00	1.76	0.43	1.40	1.04	0.87
16	0.23	0.20	0.12	0.18	0.00	6.07	0.75	1.65	0.23	0.22

## Data Availability

Data are contained within the article.
